# Development and Comparative Performance of Physiologic Monitoring Strategies in the Emergency Department

**DOI:** 10.1001/jamanetworkopen.2022.33712

**Published:** 2022-09-28

**Authors:** David Kim, Boyang Tom Jin

**Affiliations:** 1 Department of Emergency Medicine, Stanford University, Palo Alto, California; 2 Department of Computer Science, Stanford University, Palo Alto, California

## Abstract

**Importance:**

Accurate and timely documentation of vital signs affects all aspects of triage, diagnosis, and management. The adequacy of current patient monitoring practices and the potential to improve on them are poorly understood.

**Objective:**

To develop measures of fit between documented and actual patient vital signs throughout the visit, as determined from continuous physiologic monitoring, and to compare the performance of actual practice with alternative patient monitoring strategies.

**Design, Setting, and Participants:**

This cross-sectional study evaluated 25 751 adult visits to continuously monitored emergency department (ED) beds between August 1, 2020, and December 31, 2021. A series of monitoring strategies for the documentation of vital signs (heart rate [HR], respiratory rate [RR], oxygen saturation by pulse oximetry [Spo_2_], mean arterial pressure [MAP]) was developed and the strategies’ ability to capture physiologic trends and vital sign abnormalities simulated. Strategies included equal spacing of charting events, charting at variable intervals depending on the last observed values, and discrete optimization of charting events.

**Main Outcomes and Measures:**

Coverage was defined as the proportion of monitor-derived vital sign measurements (at 1-minute resolution) that fall within the bounds of nursing-charted values over the course of an ED visit (HR ± 5 beats/min, RR ± 3 breaths/min, Spo_2_ ± 2%, and MAP ± 6 mm Hg). Capture was defined as the documentation of a vital sign abnormality detected by bedside monitor (tachycardia [HR >100 beats/min], bradycardia [HR <60 beats/min], hypotension [MAP <65 mm Hg], and hypoxia [Spo_2_ <95%]).

**Results:**

Median patient age was 60 years (IQR, 43-75 years), and 13 329 visits (51.8%) were by women. Monitored visits had a median of 4 (IQR, 2-5) vital sign charting events per visit. Compared with actual practice, a simple rule, which observes vital signs more frequently if the last observation fell outside the bounds of the previous values, and using the same number of observations as actual practice, produced relative coverage improvements of 31.5% (95% CI, 30.5%-32.5%) for HR, 31.0% (95% CI, 30.0%-32.0%) for MAP, 16.8% (95% CI, 16.0%-17.6%) for RR, and 7.8% (95% CI, 7.3%-8.3%) for Spo_2_. The same strategy improved capture of abnormalities by 38.9% (95% CI, 26.8%-52.2%) for tachycardia, 38.1% (95% CI, 29.0%-47.9%) for bradycardia, 39.0% (95% CI, 24.2%-55.7%) for hypotension, and 123.1% (95% CI, 110.7%-136.3%) for hypoxia. Analysis of optimal coverage suggested an additional scope for improvement through more sophisticated strategies.

**Conclusions and Relevance:**

In this cross-sectional study, actual documentation of ED vital signs was variable and incomplete, missing important trends and abnormalities. Alternative monitoring strategies may improve on current practice without increasing the overall frequency of patient monitoring.

## Key Points

**Question:**

How well do documented vital signs reflect the physiologic trends of emergency department patients, and how can vital sign monitoring be improved to better capture clinically relevant changes?

**Findings:**

In this cross-sectional study of 25 751 monitored adult emergency department visits, documented vital signs offered a limited description of actual physiologic trends and failed to capture many vital sign abnormalities. Simulated alternative monitoring strategies were associated with significant improvement in the description of patient physiology and produced more timely alerts of clinical deterioration.

**Meaning:**

These findings suggest that current patient monitoring practices may be improved without increasing the overall frequency of patient monitoring.

## Teaser

This cross-sectional study assesses the concordance between actual patient vital signs during emergency department (ED) visits and those documented in patient medical records and evaluates strategies for improved monitoring and documentation of vital signs in the ED.

## Introduction

Vital signs (heart rate [HR], respiratory rate [RR], blood pressure, oxygen saturation, and temperature) are fundamental to patient triage, diagnosis, management, and monitoring. Because changes in vital signs often signal a change in clinical status,^1^ a patient’s vital signs are typically recorded on initial presentation and periodically throughout an emergency department (ED) visit or hospital admission. These recorded vital signs guide clinical decision-making and summarize the patient’s physiologic trajectory. In the commonly used Emergency Severity Index (ESI) triage algorithm (ranging from 1 [most urgent] to 5 [least urgent]), certain vital sign abnormalities (eg, HR >100 beats/min, RR >20 breaths/min, or oxygen saturation by pulse oximetry [Spo_2_] <92%) prompt consideration of a higher triage acuity.^2^ Accurate vital signs are also essential for posttriage clinical decision instruments and predictive models.^3,4,5^ The frequency and timing of vital sign monitoring commonly varies by location (eg, ED, hospital ward, intensive care unit), nursing resources, and patient condition.^6^ Although delayed or erroneous charting of vital signs has been associated with medical errors and adverse outcomes,^7,8^ the clinical adequacy of existing charting practices and the potential to improve on them are poorly understood.

In many settings, vital signs are obtained from bedside physiologic monitors, which record vital signs either continuously (as for HR, RR, and Spo_2_) or intermittently (as for blood pressure by sphygmomanometry). Although these data can, in principle, be stored and directly reviewed, in practice, they most often enter the electronic health record when nursing staff manually reviews the bedside monitor and records the current values. These recorded values, when erroneous or delayed in capturing clinically relevant changes in patient status, can lead to adverse outcomes.^7,8^ However, simply increasing the frequency of charting or relying directly on continuous monitoring data is unlikely to mitigate the problem. Although some EDs employ monitor technicians to improve the recording and communication of vital sign abnormalities, the time and attention of clinicians remain scarce resources, and the recording and review of redundant or uninformative vital signs may simply distract from clinically relevant changes.^9,10^

Few previous studies have directly compared continuously recorded and nursing-charted vital signs. One study found substantial discrepancies between nursing documentation and bedside monitors in the detection of apnea in infants.^11^ Studies of anesthesia records found inaccuracies in handwritten compared with computerized records^12^ and resistance to documenting extreme or suddenly changing values.^13^ To our knowledge, no study has quantified the global accuracy of vital sign recording or evaluated potential strategies to improve it.

In this article, we introduce the concepts of vital sign coverage and capture. Coverage denotes the proportion of actual vital signs, derived from continuous monitoring, that fall within the bounds of nurse-entered values. Capture refers to the detection of specific abnormalities (tachycardia, bradycardia, hypotension, and hypoxia) and the delay from occurrence to charting. We studied 25 751 visits to monitored beds of 1 academic ED and calculated vital sign coverage and capture. We developed an optimized charting strategy, using methods from discrete optimization, and a suite of prospective rule-based strategies and compared their performance with actual charting by ED nursing staff. We conclude with implications for automated and hybrid approaches to patient monitoring.

## Methods

### Data Sources and Transformations

In this cross-sectional study, we analyzed 25 751 visits to continuously monitored beds of the Stanford Health Care Emergency Department between August 1, 2020, and December 31, 2021. For each visit, we observed patient age, sex, triage acuity by ESI, and charted vital signs from ED triage to ED departure, including HR, RR, Spo_2_, and systolic blood pressure (SBP) and diastolic blood pressure (DBP) summarized as mean arterial pressure (*MAP* = [1/3]*SBP* + [2/3]*DBP*). We obtained corresponding vital sign measurements (HR, RR, Spo_2_, and MAP) from IntelliVue bedside monitors (Koninklijke Philips). To reduce the effect of localized variation and noise, we used the 1-minute mean of each measurement. Where measurements of a given modality were missing in the record, we carried the last observation forward. Because MAP was obtained through intermittent sphygmomanometry, intervals between measurements were set to the last recorded measurement. This study followed the Strengthening the Reporting of Observational Studies in Epidemiology (STROBE) reporting guideline. The study was approved by the institutional review board of Stanford University, with a waiver of consent for retrospective research on anonymized data.

### Coverage of Vital Sign Trends

**Figure 1.**
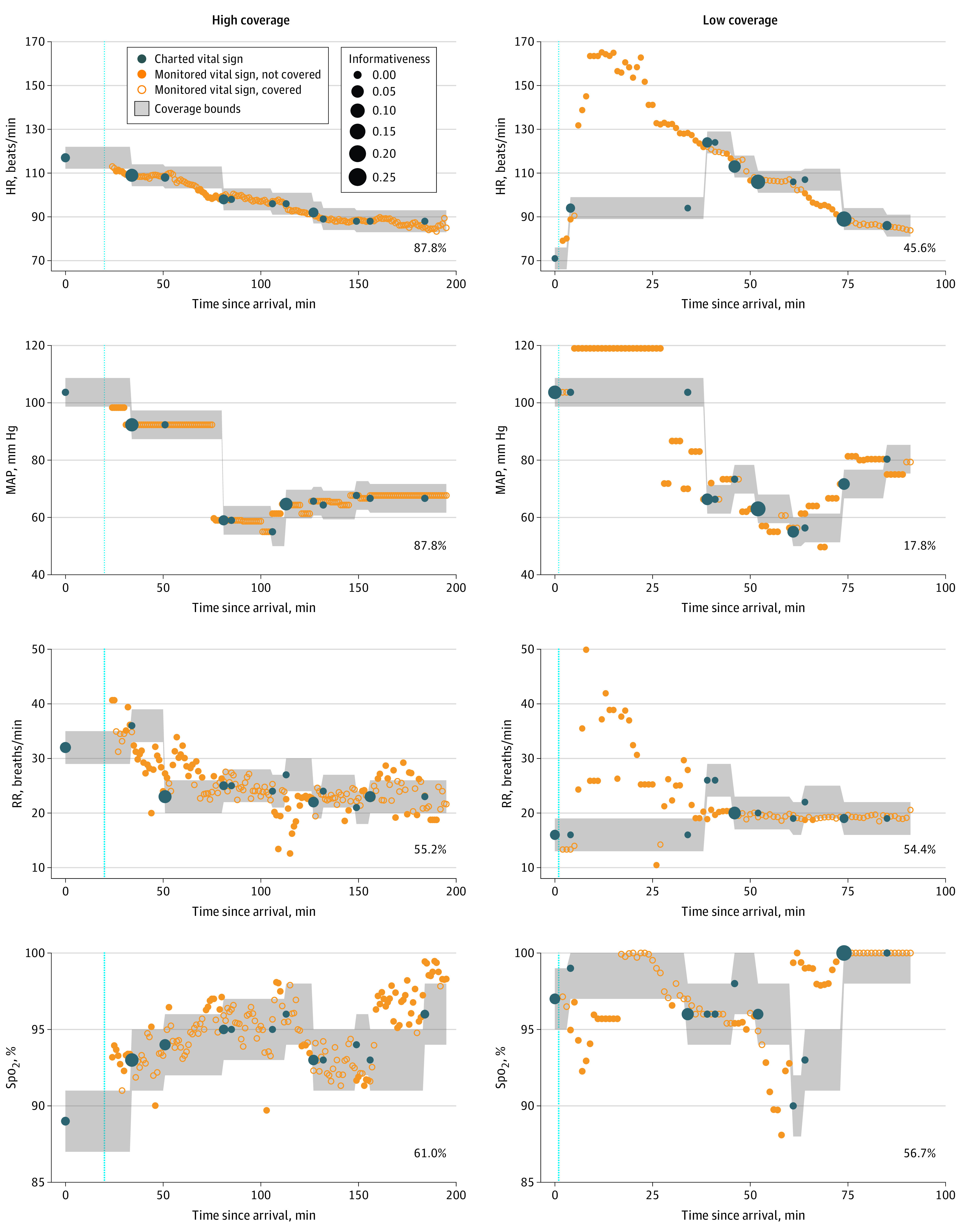
Emergency Department Visits With High and Low Vital Sign Coverage Coverage is the proportion of monitored vital signs that fall within the bounds of charted values (bottom right of each panel). Monitored vital signs represent 1-minute means of recorded values. Point size of charted vital signs is proportional to informativeness (ie, the reduction in coverage of that modality if that observation is removed). HR indicates heart rate; MAP, mean arterial pressure; RR, respiratory rate; Spo_2_, oxygen saturation by pulse oximetry. Dashed blue line indicates initial rooming time.

Coverage is defined as the proportion of monitor-derived vital sign measurements (at 1-minute resolution) that fall within the bounds of nursing-charted values over the course of an ED visit. A monitor-derived vital sign is covered if it falls within the bounds for the corresponding modality as follows: HR ± 5 beats/min, RR ± 3 breaths/min, Spo_2_ ± 2%, and MAP ± 6 mm Hg. These values represent the mean SD of each vital sign across monitored visits, rounded to the nearest integer. Coverage bounds are carried forward until the next nursing observation. Figure 1 illustrates the calculation of vital sign coverage for 2 ED visits with comparable overall charting frequency (3.7 and 3.9 charting events/h, respectively). The high-coverage visit (HR, 87.8%; MAP, 87.2%; RR, 55.2%; Spo_2_, 61.0%) exhibits nursing documentation that closely matches the patient’s evolving physiology, including a decrease in blood pressure, whereafter charting frequency is increased. The low-coverage visit (HR, 45.6%; MAP, 17.8%; RR, 54.4%; Spo_2_, 56.7%) exhibits poorer overall fit between charted and monitored vital sign trends, with gaps in charting during which the patient exhibits substantial changes in vital signs, delayed recognition of decreased blood pressure, and several uninformative charting events that do not provide new information. Informativeness reflects the contribution of a specific charting event, namely, the reduction in coverage of that modality if that observation is removed.

### Vital Sign Monitoring Strategies

We developed 4 monitoring strategies with which actual practice was compared. At the study hospital, ED nurses are instructed to record vital signs approximately once per hour, with more frequent charting as needed to reflect interventions or changes in patient status.

#### Equal Spacing Strategy

The equal spacing strategy distributes the same number of nursing charting events evenly across the visit. This strategy assumes knowledge of the total number of charting events in a visit but not of vital signs themselves.

#### Fixed Schedule Strategy

The fixed schedule strategy is prospective and assumes no knowledge of the visit. In this strategy, vital sign charting occurs at the start of monitoring, then at the median interval between charting events for a given ESI at triage (ESI 1, 13 minutes; ESI 2, 45 minutes; ESI 3, 71 minutes; and ESI 4, 65 minutes).

#### Variable Schedule Strategy

The variable schedule strategy is also prospective. The first charting event occurs after the monitor registers the first values of at least 2 vital signs. If all vital signs at a given observation fall within the coverage bounds of the previous observation (for the first charting event after rooming, the previous observation may be in triage), the patient is assumed to be stable, and the next observation is made after a large step (we evaluated values from 20 to 120 minutes). If any of the vital signs at a given observation fall outside the bounds of the previous observation, the patient is assumed to exhibit evolving physiology, and the next charting observation is made after a small step (we evaluated values from 10 to 90 minutes). We used a grid search strategy to evaluate combinations of small and large steps, calculating the resulting vital sign coverage and the number of charting events produced by each strategy. We selected for further evaluation the step values producing the expected number of charting events closest to but not exceeding actual practice.

#### Optimized Strategy

The optimized strategy finds a possible placement of charting events in order to maximize coverage of patient vital sign trends, using the same number of actual nursing charting events or fewer. This strategy assumes knowledge of the entire visit and places an upper bound on the maximal achievable coverage of vital signs with a given number of charting events. The eMethods in the Supplement describes the method in detail. This strategy was implemented with dynamic programming in Python, version 3.8^14^ (Python Software Foundation), enumerating all possible configurations of charting events and selecting 1 sequence of charting events to maximize overall coverage across all modalities.^15^

**Figure 2.**
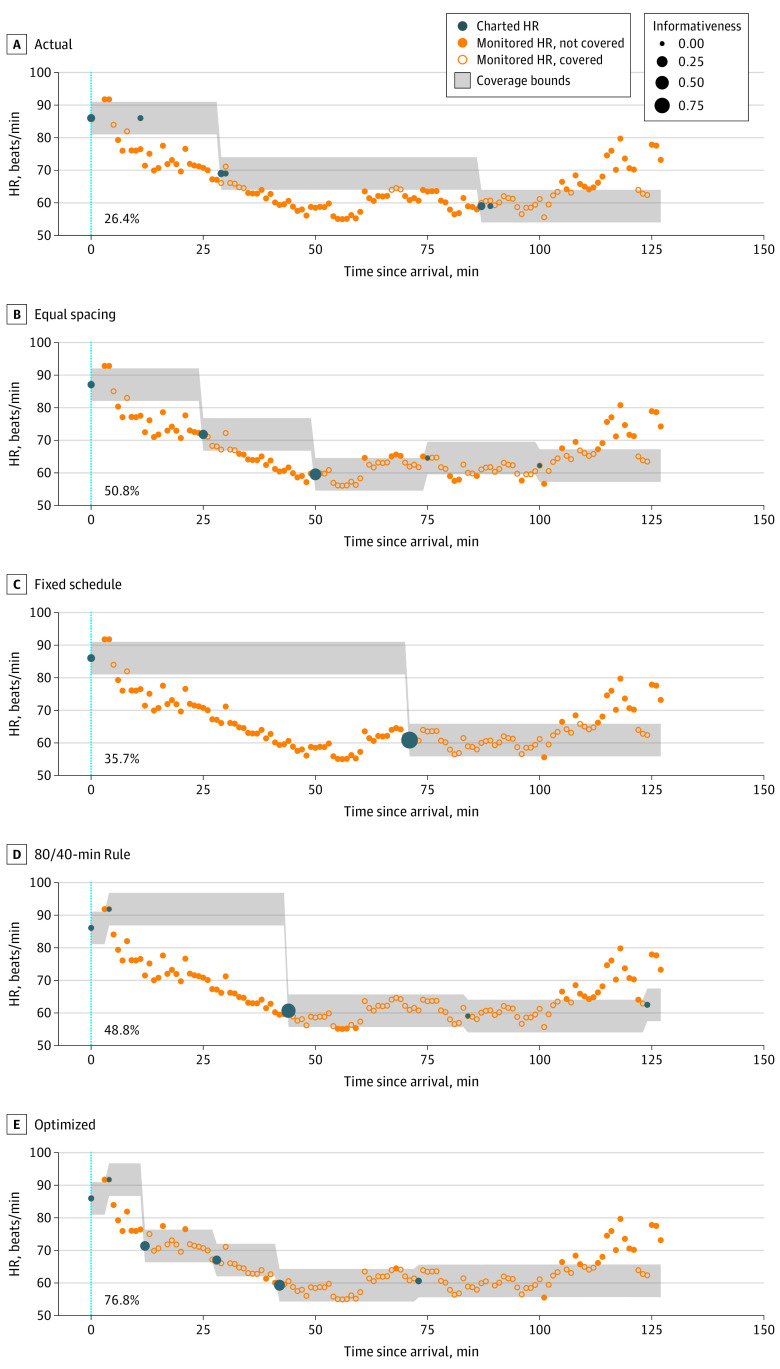
Comparison of Heart Rate (HR) Coverage for Actual Charting and Four Alternative Strategies Coverage is the proportion of monitored HR values that fall within the bounds of charted values (bottom left of each panel). Monitored HR values represent 1-minute means of recorded values. Informativeness is the proportional reduction in coverage incurred if a given observation is removed. The alternative charting strategies outperformed actual charting by varying degrees and produced individually more informative observations. The variable schedule (80/40-minute rule) and optimized strategies depend on values of other vital signs (not shown). Dashed blue lines indicate initial rooming time.

We calculated the visit- and modality-specific coverage produced by each strategy and compared coverage for each strategy against coverage achieved by actual nursing practice using 2-sample *t* tests. We calculated relative improvement in coverage over baseline practice using *t* tests for ratios of means with heterogeneous group variances.^16^ Figure 2 illustrates HR coverage by actual charting and each alternative strategy.

### Capture of Vital Sign Abnormalities

We evaluated the performance of actual charting and each of the 4 simulated alternative charting strategies in capturing the following vital sign abnormalities detected by bedside monitor: tachycardia (HR >100 beats/min), bradycardia (HR <60 beats/min), hypotension (MAP <65 mm Hg), and hypoxia (Spo_2 _<95%). Specifically, we defined an event as an abnormality that arises after initiation of monitoring (ie, not present in the first 5 minutes of monitoring). An event is captured if charting subsequently notes the same abnormality. For captured events, we calculated the time in minutes from appearance on the monitor to capture in charting. We compared the proportion of abnormal events captured by each alternative strategy to actual practice using 2-sample χ^2^ tests with continuity correction. We calculated 95% CIs for the ratios of proportions using a skewness-corrected likelihood score–based method.^17^ For captured events, we compared time to capture against actual practice using Wilcoxon signed rank tests and calculated 95% CIs of differences in median time to capture using bootstrap resampling with 10 000 replicates.

Statistical tests were performed using R, version 4.1 software (R Foundation for Statistical Computing). All tests were 2-sided with an a priori significance threshold of *P* < .05.

## Results

### Visit Characteristics

Of 77 575 adult ED visits to monitored beds during the study period, 25 751 visits (33.2%) had values for each of HR, MAP, RR, and Spo_2_ both charted by nursing and recorded and stored by bedside monitor. A total of 35 161 visits (45.3%) were excluded because of the absence of 1 or more monitored vital signs, and a further 16 663 (21.5%) were excluded because of absence of 1 or more charted vital signs. Among fully monitored and charted visits, the median patient age was 60 years (IQR, 43-75 years); 13 329 visits (51.8%) were by women and 12 422 (48.2%) by men. Of the monitored visits, 510 (2.0%) were triaged at ESI 1, 9089 (35.3%) at ESI 2, 15 835 (61.5%) at ESI 3, 317 (1.2%) at ESI 4, and 0 at ESI 5. Monitored visits had a median rooming-to-departure time of 324 minutes (IQR, 233-439 minutes) and a median of 4 (IQR, 2-5) vital sign charting events per visit. Discharge home from the ED occurred in 13 013 (50.5%) visits. eTable 1 in the Supplement lists detailed visit characteristics. eTable 2 in the Supplement compares characteristics of included (fully monitored and charted) and excluded visits. Compared with excluded visits, patients with monitored visits were older, more likely to be men, were more likely to be triaged at ESI 1 and 2, had longer lengths of stay, and were less likely to be discharged home from the ED.

### Selection of Variable Schedule Strategy

**Figure 3.**
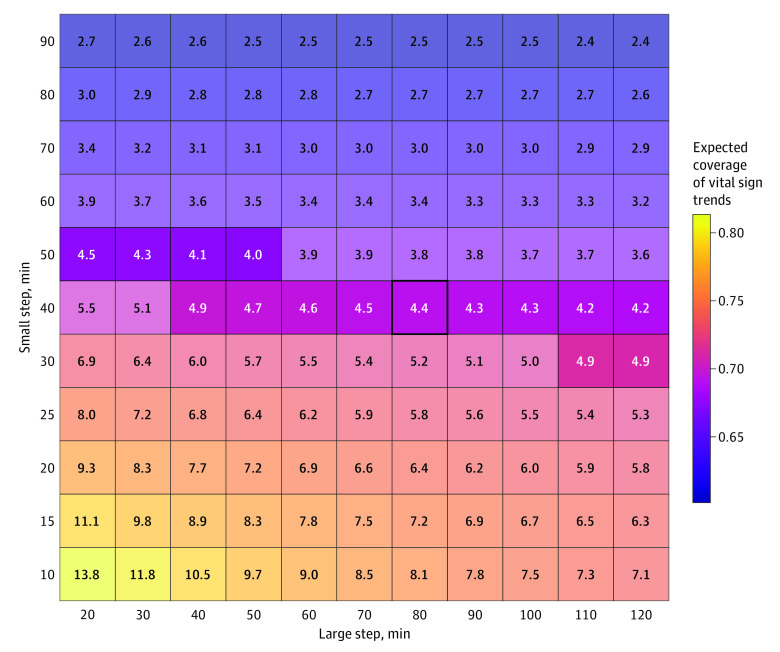
Performance of Variable Schedule Strategies in Capturing Vital Sign Trends Each strategy entails charting vital signs at the large-step interval and switching to the small-step interval when 1 or more observed vital signs falls outside the bounds dictated by the previous observation. Fill color gives expected mean coverage across heart rate, mean arterial pressure, respiratory rate, and oxygen saturation by pulse oximetry. Each cell shows the mean number of charting events per visit produced by each strategy. The selected strategy (bold borders) is the 80/40-minute rule, which produces the number of charting events closest to but not exceeding actual charting practice (4.4 observations/visit). The highlighted strategies fall within ±0.5 observations/visit.

The equal spacing, fixed schedule, and optimized charting strategies each produced a single policy. By contrast, we evaluated 121 possible variable schedule strategies reflecting combinations of 11 possible large steps (from 20 to 120 minutes) and 11 possible small steps (from 10 to 90 minutes). Figure 3 shows the mean coverage (across all vital signs) as well as the mean number of charting events per visit produced by each policy. We selected for further evaluation the policy producing the expected number of charting events closest to, but not exceeding, actual practice as follows: 80-minute large step and 40-minute small step (80/40-minute rule), which produced a mean (SD) of 4.4 (2.1) vital sign charting events per visit (compared with 4.5 in actual practice). The eFigure in the Supplement gives an alternative depiction of the relationship between coverage and charting events under variable schedule strategies. Performance characteristics of these strategies were determined substantially by the size of the small step, and most variable schedule strategies with small steps of 40 minutes or longer produced better overall coverage with fewer charting events than actual practice.

### Distribution of Charting Events

**Figure 4.**
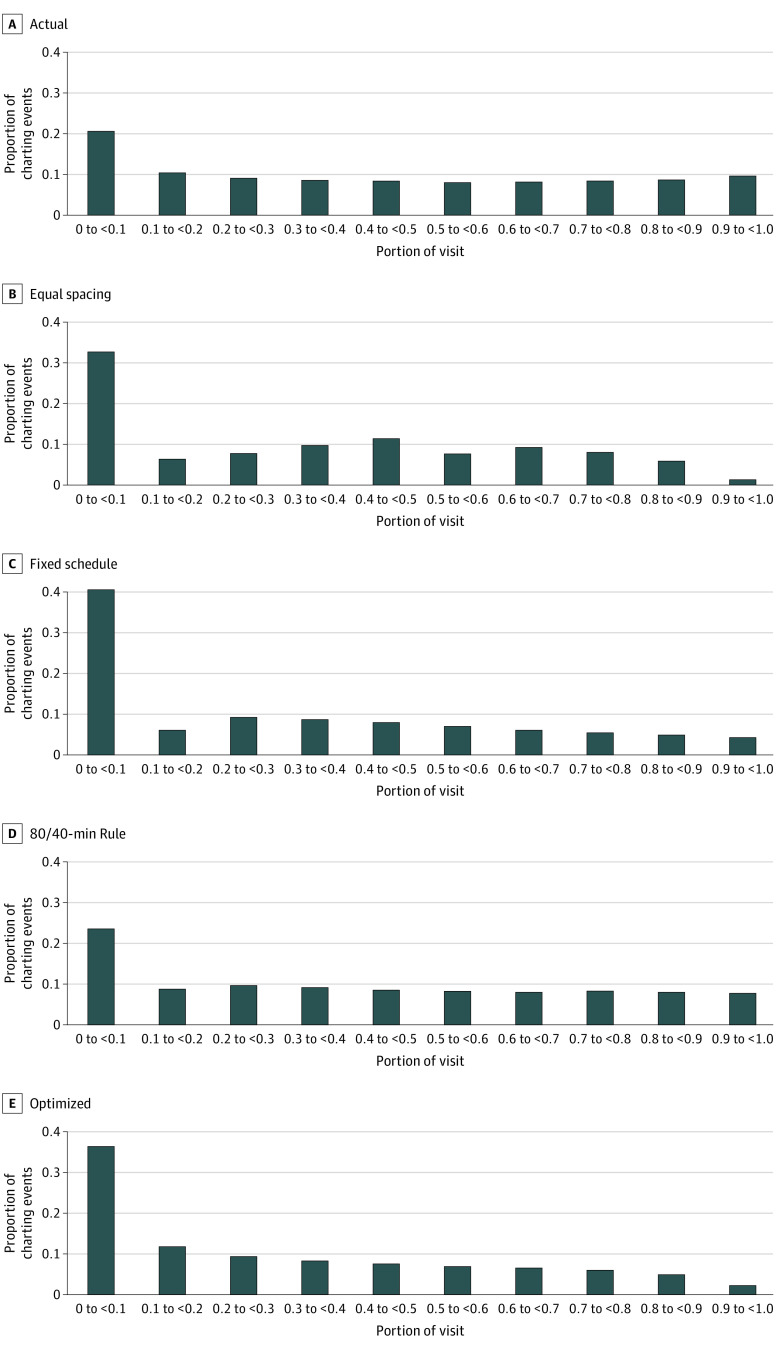
Distribution of Charting Events Over the Visit by Charting Strategy Histograms show the proportion of charting events falling within each tenth of the monitored emergency department visit. All strategies favor increased observation of vital signs at the start of the visit. Optimized charting is more biased toward the earlier portion of the visit than actual practice. The equal spacing and fixed schedule strategies are not uniform in this aggregate visualization because the number of charting events, and therefore the spacing of events, is highly variable across visits.

Figure 4 shows the distribution of charting events produced under each of the 5 charting strategies: actual practice, equal spacing, fixed schedule, 80/40-minute rule, and optimized charting. All strategies favor increased observation of vital signs at the start of the visit. Optimized charting is more biased toward the earlier portion of the visit than actual practice and is unique among charting strategies in favoring steadily declining charting frequency over the course of the ED visit.

### Coverage of Vital Sign Trends and Capture of Abnormal Vital Signs

**Figure 5.**
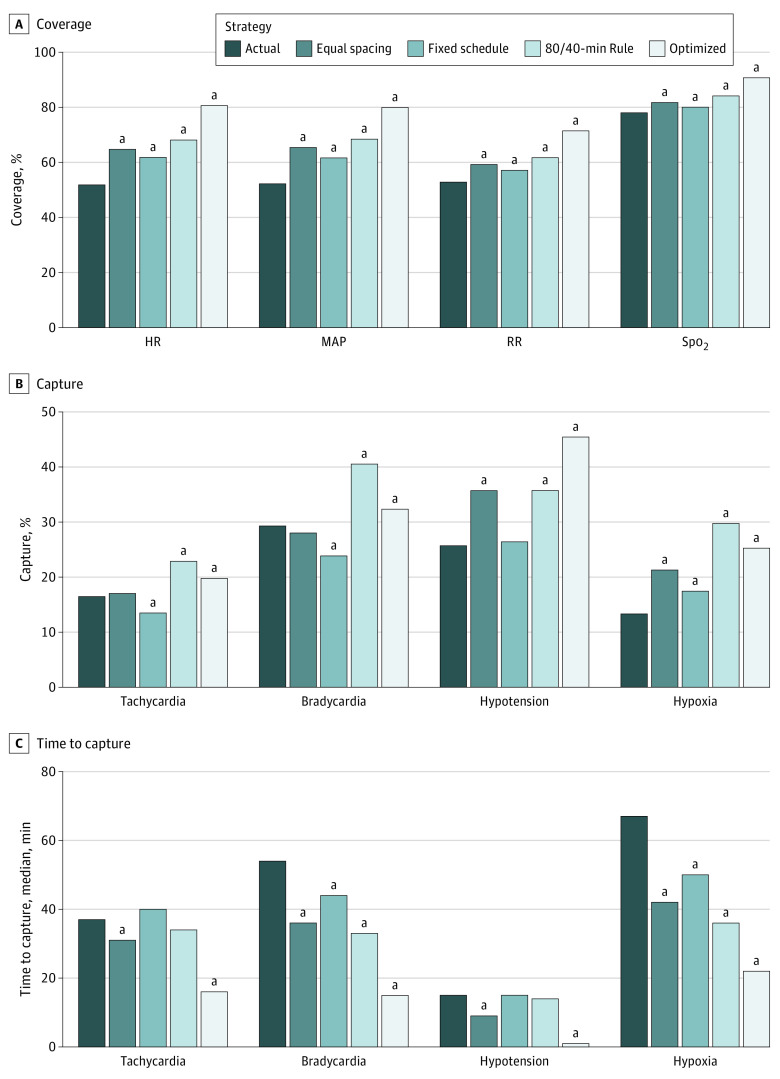
Comparative Performance of Vital Sign Charting Strategies HR indicates heart rate; MAP, mean arterial pressure; RR, respiratory rate; Spo_2_, oxygen saturation by pulse oximetry. ^a^Differences from actual charting significant at *P* < .05. eTable 3 in the Supplement shows performance differences compared with actual charting and the 95% CIs of each difference.

Figure 5 and eTable 3 in the Supplement show the performance of actual nursing practice and each of the 4 alternative charting strategies (equal spacing, fixed schedule, 80/40-minute rule, and optimized charting). In actual practice, vital sign charting achieved mean (SD) coverage of 51.8% (27.5%) for HR, 52.2% (28.1%) for MAP, 52.8% (23.8%) for RR, and 78.0% (23.3%) for Spo_2_. For all vital signs, all 4 alternative strategies produced superior coverage over actual charting (all comparisons, *P* < .001 by 2-sample *t* test). Optimized charting produced the highest coverage of vital sign trends, followed by the 80/40-minute rule and then by equal spacing and fixed scheduling of charting events. Unlike equal spacing and optimal charting, the fixed schedule and 80/40-minute rule strategies presume no knowledge of the visit and could be deployed prospectively. Of the 2 prospective strategies, the 80/40-minute rule was more performant. Compared with actual practice, this rule, producing the same expected number of charting events per visit, achieved relative coverage improvements of 31.5% (95% CI, 30.5%-32.5%) for HR, 31.0% (95% CI, 30.0%-32.0%) for MAP, 16.8% (95% CI, 16.0%-17.6%) for RR, and 7.8% (95% CI, 7.3%-8.3%) for Spo_2_.

Across the 25 751 monitored ED visits, 3888 patients (15.1%) developed tachycardia (HR >100 beats/min), 3192 (12.4%) developed bradycardia (HR <60 beats/min), 1417 (5.5%) developed hypotension (MAP <65 mm Hg), and 10 346 (40.2%) developed hypoxia (Spo_2_ <95%). Actual charting documented 640 (16.5%) of 3888 patients with new tachycardia at a median lag of 37 minutes (IQR, 10-90 minutes), 936 (29.3%) of 3192 with new bradycardia at a median lag of 54 minutes (IQR, 20-107 minutes), 364 (25.7%) of 1417 with new hypotension at a median lag of 15 minutes (IQR, 4-40 minutes), and 1379 (13.3%) of 10 346 with new hypoxia at a median lag of 67 minutes (IQR, 25-132 minutes). For capture of these events, the 80/40-minute rule was overall the most performant alternative charting strategy, achieving the following relative improvements over actual practice in the capture of new vital sign abnormalities: 38.9% (95% CI, 26.8%-52.2%) for tachycardia, 38.1% (95% CI, 29.0%-47.9%) for bradycardia, 39.0% (95% CI, 24.2%-55.7%) for hypotension, and 123.1% (95% CI, 110.7%-136.3%) for hypoxia. Among captured events, the 80/40-minute rule had a 31-minute shorter median time to capture of hypoxia (95% CI of difference, 25-37 minutes) and a 21-minute shorter median time to capture of bradycardia (95% CI of difference, 14-27 minutes) compared with actual practice.

eTable 3 in the Supplement lists the absolute performance differences compared with actual charting and the 95% CIs of each difference for each vital sign, monitoring strategy, and performance measure. eTable 4 in the Supplement lists coverage and capture measures separately for visits triaged at ESI 2 and 3, which collectively account for 24 924 monitored visits (96.8%). eTable 5 in the Supplement lists coverage for ESI 2 visits with and without vital sign abnormalities at triage. In general, visits with abnormalities at triage had poorer coverage of vital signs other than MAP. More performant monitoring strategies (eg, 80/40-minute rule) showed smaller coverage discrepancies between visits with and without abnormalities at triage.

## Discussion

Vital signs are among the most important patient data in medicine, and the timeliness and accuracy of their documentation affect all aspects of triage, diagnosis, and management. Although some authors have proposed ESI-based guidance for the frequency of vital sign documentation,^18^ no generally accepted guidelines exist.^6,18^ In this cross-sectional study, we used high-resolution continuous patient monitoring data to assess the quality of vital sign charting, specifically the ability of charted vital signs to accurately describe trends in vital signs throughout a visit and to capture potentially relevant abnormalities. We found substantial potential to improve the documentation of vital signs in the ED and showed that alternative charting strategies were associated with a significant improvement in the informativeness of vital sign documentation without requiring more frequent patient monitoring or increasing the number of data points that a clinician would face.

Vital signs are ubiquitous in clinical decision instruments and, increasingly, are tracked over time to generate warnings of clinical decompensation.^19,20,21^ Where the bedside monitors from which vital signs are collected are integrated into the electronic health record, the recording of vital signs can be automated in whole or in part. Vital signs and their trends, however, still must be reviewed and acted on by clinicians with limited time and attention, raising the need for a parsimonious and accurate record of a patient’s physiologic trajectory over the course of a visit. Our analysis suggests that monitoring could be improved by observing patients more frequently early in their visits, when physiology is more likely to be labile and resuscitative measures are not yet complete. Simple prospective rules, whereby patients are observed at shorter intervals if the last observation falls outside predefined bounds of clinical stability, can improve the coverage of patient trends and the capture of specific abnormalities, though in some cases, strategies may exhibit trade-offs between these related but distinct objectives.

By quantifying the fit between charted and actual vital signs, we hope to provide a conceptual framework for efforts to improve vital sign documentation, maximize the efficiency of nursing and clinician behavior, and design human-machine interfaces that enhance the information available to clinicians while avoiding information overload and alarm fatigue.^9,10^

### Limitations

This study has several limitations. We studied visits from a single academic ED, with nursing resources and practices not representative of all care settings. For instance, our study ED was fortunate to have experienced fewer COVID-19–related staff shortages than many other hospitals. The rarity of permanently stored bedside monitor data makes multicenter analysis difficult. Our study population (patients in whom all vital signs were recorded both by nursing staff and by monitor) was older and had more acute conditions than the overall ED population, with the majority of included visits triaged at ESI 2 and 3, limiting inference about higher- or lower-acuity visits. We speculate, however, that the fit of charted to actual vital signs may be less important for resuscitations, which are closely monitored, and for low-acuity visits, where patients are less likely to exhibit physiologic changes requiring timely documentation.

Our definition of coverage and the selection of acceptable error bounds permit many possible variations. Equal weighting of all vital signs may not be appropriate in all settings. Our definition of abnormality capture is likewise one among many possible formulations, and not all vital sign abnormalities need to be recorded. Many are likely too transient to be clinically meaningful, though even intermittent or transient abnormalities can have prognostic value.^22,23^ Although we analyzed 1-minute means of monitored vital signs to reduce the effect of artifacts, false alarms may remain, affecting the accuracy of capture rates. The rate of at least transient hypoxia (Spo_2_ <95%) in our study cohort is high (40.2%), which may reflect such false alarms, as well as high rates of true hypoxia owing to collection of data during the COVID-19 pandemic. We report relative in addition to absolute differences in coverage and capture across charting strategies to mitigate dependence on specific definitions.

We report on only 4 of many possible monitoring strategies. For instance, our variable schedule strategy (80/40-minute rule) prompts repeat observation at a shorter interval when the current observation falls outside the bounds of the previous observation, irrespective of whether these observations fall within the normal range. An alternative strategy might instead, or additionally, prompt more frequent observation in response to abnormal values. Finally, although delayed or inaccurate charting of vital signs is known to be associated with adverse outcomes,^7,8^ understanding the practical and clinical implications of specific patient monitoring strategies will require analysis of provider behavior in response to recorded vital signs and prospective evaluation.

## Conclusions

Although optimal monitoring practices will vary by clinical setting and available resources, our findings in this cross-sectional study suggest that current practice makes inefficient use of nursing and clinician attention. Simple charting strategies have the potential to improve the informativeness of patient monitoring. These strategies await prospective, multicenter evaluation and assessment of an association with operational and clinical outcomes.

## Notes

Supplement. **eMethods.** Optimized Coverage Via a Discrete Optimization Approach **eTable 1.** Visit Characteristics Overall and by Emergency Severity Index at Triage **eTable 2.** Visit Characteristics of Included and Excluded Visits **eFigure.** Clustering of Variable Schedule Strategies by Small Step Size **eTable 3.** Performance Comparison of Vital Sign Charting Strategies **eTable 4.** Performance of Vital Sign Charting Strategies by Emergency Severity Index **eTable 5.** Mean Coverage Performance of Vital Sign Charting Strategies for Emergency Severity Index Level 2 Visits With or Without Abnormalities at Triage
