# Supplementary material for: Development and Comparative Performance of Physiologic Monitoring Strategies in the Emergency Department
Source: JAMA Netw Open. 2022 Sep 28;5(9):e2233712. doi: 10.1001/jamanetworkopen.2022.33712 (PMC9520367; doi:10.1001/jamanetworkopen.2022.33712)
Supplement: Supplement. — eMethods. Optimized Coverage Via a Discrete Optimization Approach eTable 1. Visit Characteristics Overall and by Emergency Severity Index at Triage eTable 2. Visit Characteristics of Included and Excluded Visits eFigure. Clustering of Variable Schedule Strategies by Small Step Size eTable 3. Performance Comparison of Vital Sign Charting Strategies eTable 4. Performance of Vital Sign Charting Strategies by Emergency Severity Index eTable 5. Mean Coverage Performance of Vital Sign Charting Strategies for Emergency Severity Index Level 2 Visits With or Without Abnormalities at Triage [file jamanetwopen-e2233712-s001.pdf]

Supplemental Online Content

Kim D, Jin BT. Development and comparative performance of physiologic monitoring strategies in the emergency department. *JAMA Netw Open*. 2022;5(9):e2233712. doi:10.1001/jamanetworkopen.2022.33712

eMethods. Optimized Coverage Via a Discrete Optimization Approach
eTable 1. Visit Characteristics Overall and by Emergency Severity Index at Triage
eTable 2. Visit Characteristics of Included and Excluded Visits
eFigure. Clustering of Variable Schedule Strategies by Small Step Size
eTable 3. Performance Comparison of Vital Sign Charting Strategies
eTable 4. Performance of Vital Sign Charting Strategies by Emergency Severity Index
eTable 5. Mean Coverage Performance of Vital Sign Charting Strategies for Emergency Severity Index Level 2 Visits With or Without Abnormalities at Triage

This supplemental material has been provided by the authors to give readers additional information about their work.

© 2022 Kim D et al. *JAMA Network Open.*

eMethods. Optimized Coverage Via a Discrete Optimization Approach

The discrete optimization problem can be formulated as follows:

$$\mathrm{C} = \max_{t_1 \ldots t_n} \frac{1}{\sum_{d \in M} |V^{(d)}|} \sum_{d \in M} \sum_{i=1}^{n} \left| \left\{ \mathbb{1}[(v_{t_i}^{(d)} - e^{(d)}) \leq v_k^{(d)} \leq (v_{t_i}^{(d)} + e^{(d)})] \;\middle|\; t_i \leq k < t_{i+1} \right\} \right|$$
$$\text{s.t.} \quad n \leq N$$

This equation maximizes coverage C across modalities M by finding one possible placement of simulated charting events at times $t_1 \ldots t_n$ such that the number of events n does not exceed the number of actual nursing charting events N.

The body of this equation describes the formula for coverage, where $V^{(d)}$ is the set of monitor events for modality d, $v_x^{(d)} \in V^{(d)}$ is the monitor event at time x, $e^{(d)}$ is the coverage bounds (HR±5, RR±3, SpO2±2, MAP±6), and k refers to the monitor event times that fall between two consecutive optimal placement times. Coverage can be calculated as follows:

1. For each monitor event $v_k^{(d)}$, consider whether it falls within the coverage bounds of the preceding simulated event $v_{t_i}^{(d)}$. This is denoted by the 𝟙 indicator function.
2. Sum the number of monitor events that satisfies the indicator function. This is denoted by $\sum_{i=1}^{n} |\{\ldots\}|$
3. Sum the preceding sum across all modalities to find the overall sum. This is denoted by $\sum_{d\,\in M}$
4. Find the coverage by dividing the overall sum by the total number of monitor events across all modalities. This is denoted by $\frac{1}{\sum_{d \epsilon M} |V^{(d)}|}$

© 2022 Kim D et al. *JAMA Network Open.*

eTable 1. Visit Characteristics Overall and by Emergency Severity Index at Triage

	**Full cohort (n = 25,751)**	**ESI = 1 (n = 510)**	**ESI = 2 (n = 9,089)**	**ESI = 3 (n = 15,835)**	**ESI = 4 (n = 317)**
Age in years, median [IQR]	60 [43-75]	66 [48-79]	61 [43-76]	60 [42-74]	55 [36-69]
Female, n (%)	13,329 (51.8%)	218 (42.7%)	4,383 (48.2%)	8,579 (54.2%)	149 (47%)
Triage VS, median [IQR]					
SpO2, %	99 [97-100]	98 [94-100]	98 [97-100]	99 [97-100]	99 [98-100]
Resp. Rate	18 [16-20]	22 [18-27]	18 [17-21]	18 [16-19]	18 [16-18]
Heart Rate	86 [74-101]	95 [75-116]	91 [76-109]	84 [73-97]	83 [73-94]
Systolic BP	136 [121-151]	135 [111-156]	135 [119-151]	136 [122-151]	134 [118-148]
Diastolic BP	79 [69-90]	82 [67-99]	80 [69-91]	79 [69-89]	79 [69-88]
MAP	98 [88-109]	102 [82-116]	98 [87-110]	98 [88-109]	97 [89-106]
ED length-of-stay, hours, median [IQR]	5.4 [3.9-7.3]	5.2 [3.7-7.0]	5.7 [4.2-7.6]	5.3 [3.7-7.2]	4.4 [2.8-6.1]
Charting events, median [IQR]	4 [2-5]	7 [4-12]	4 [3-7]	3 [2-5]	3 [2-4]
Discharged from ED, n (%)	13,013 (50.5%)	46 (9.0%)	3,276 (36.0%)	9,434 (59.6%)	257 (81.1%)

© 2022 Kim D et al. *JAMA Network Open.*

eTable 2. Visit Characteristics of Included and Excluded Visits

	**Included (n = 25,751)**	**Excluded (n = 51,824)**	**p-value of difference**
Age in years, median [IQR]	60 [43-75]	49 [33-66]	< 0.001
Female, n (%)	13,329 (51.8%)	28,860 (55.7%)	< 0.001
Triage VS, median [IQR]			
SpO2, %	99 [97-100]	99 [98-100]	< 0.001
Resp. Rate	18 [16-20]	18 [16-18]	< 0.001
Heart Rate	86 [74-101]	84 [73-96]	< 0.001
Systolic BP	136 [121-151]	134 [121-148]	< 0.001
Diastolic BP	79 [69-90]	78 [69-88]	< 0.001
MAP	98 [88-109]	97 [88-107]	< 0.001
ESI, n (%)			
1-Resuscitation	510 (2.0%)	293 (0.6%)	< 0.001
2-Emergent	9,089 (35.3%)	8,399 (16.2%)	< 0.001
3-Urgent	15,835 (61.5%)	35,268 (68.1%)	< 0.001
4-Semi-urgent	317 (1.2%)	6,976 (13.5%)	< 0.001
5-Non-urgent	0 (0.0%)	492 (0.9%)	< 0.001
Not recorded	0 (0.0%)	396 (0.8%)	< 0.001
ED length-of-stay, hours, median [IQR]	5.4 [3.9-7.3]	4.1 [2.6-6.2]	< 0.001
Discharged from ED, n (%)	13,013 (50.5%)	36,314 (70.1%)	< 0.001

Excluded visits lacked charted or monitor-recorded data for one or more vital signs (HR, MAP, RR, and SpO2). Continuous variables are compared with Wilcoxon rank-sum tests, proportions with two-sided chi-squared tests.

© 2022 Kim D et al. *JAMA Network Open.*

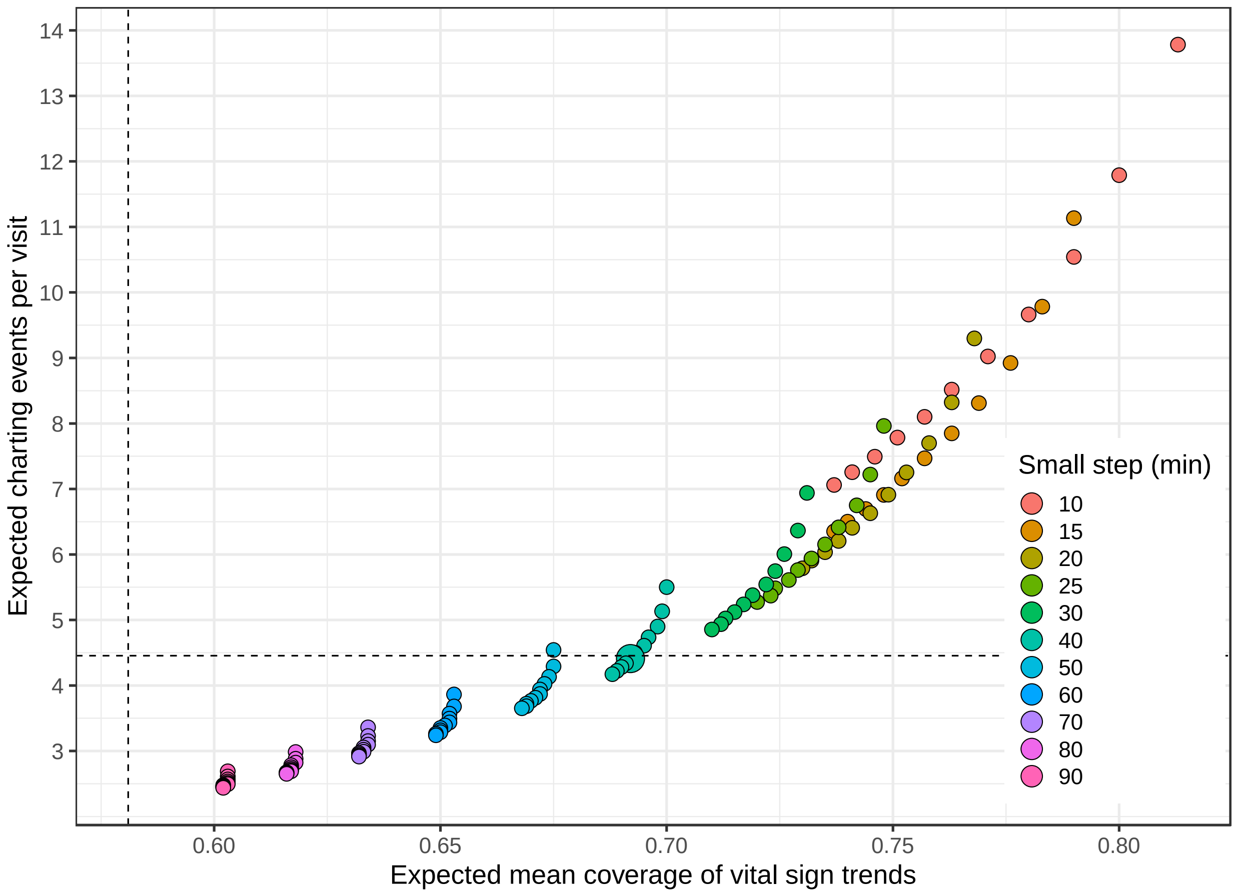

eFigure. Clustering of Variable Schedule Strategies by Small Step Size**.** Dashed lines give mean coverage across modalities (58.1%), and mean charting events per visit (4.4) in actual practice.

© 2022 Kim D et al. *JAMA Network Open.*

eTable 3. Performance Comparison of Vital Sign Charting Strategies

		**Actual**	**Equal spacing**		**Fixed schedule**		**80min-40min rule**		**Optimized**	
		Value	Value	*Diff. from actual (95% CI)*	Value	*Diff. from actual (95% CI)*	Value	*Diff. from actual (95% CI)*	Value	*Diff. from actual (95% CI)*
**VS coverage, mean (SD)**	HR	51.8% (27.5%)	64.7% (25.1%)	*+12.9% (12.4%, 13.3%)*	61.8% (26.1%)	*+9.9% (9.5%, 10.4%)*	68.1% (25.1%)	*+16.3% (15.9%, 16.7%)*	80.6% (16.6%)	*+28.8% (28.4%, 29.2%)*
	MAP	52.2% (28.1%)	65.4% (25.5%)	*+13.2% (12.8%, 13.7%)*	61.6% (26.7%)	*+9.4% (9.0%, 9.9%)*	68.4% (25.5%)	*+16.2% (15.7%, 16.6%)*	79.9% (18.2%)	*+27.8% (27.4%, 28.2%)*
	RR	52.8% (23.8%)	59.2% (23.0%)	*+6.4% (6.0%, 6.8%)*	57.1% (23.9%)	*+4.3% (3.9%, 4.7%)*	61.7% (23.0%)	*+8.9% (8.5%, 9.2%)*	71.4% (16.7%)	*+18.6% (18.2%, 18.9%)*
	SpO2	78.0% (23.3%)	81.7% (21.6%)	*+3.7% (3.3%, 4.1%)*	80.0% (23.3%)	*+1.9% (1.5%, 2.3%)*	84.1% (21.6%)	*+6.1% (5.7%, 6.4%)*	90.7% (12.4%)	*+12.6% (12.3%, 12.9%)*
**Proportion of abnormal events captured**	Tachycardia (HR > 100)	16.5% (640/3888)	17.1% (663/3888)	*+0.6% (-1.1%, 2.3%)*	13.5% (525/3888)	*-3.0% (-4.6%, -1.3%)*	22.9% (889/3888)	*+6.4% (4.6%, 8.2%)*	19.8% (769/3888)	*+3.3% (1.6%, 5.1%)*
	Bradycardia (HR < 60)	29.3% (936/3192)	28.0% (895/3192)	*-1.3% (-3.5%, 1.0%)*	23.8% (761/3192)	*-5.5% (-7.7%, -3.3%)*	40.5% (1293/3192)	*+11.2% (8.8%, 13.5%)*	32.3% (1032/3192)	*+3.0% (0.7%, 5.3%)*
	Hypotension (MAP < 65)	25.7% (364/1417)	35.7% (506/1417)	*+10.0% (6.6%, 13.5%)*	26.4% (374/1417)	*+0.7% (-2.6%, 4.0%)*	35.7% (506/1417)	*+10.0% (6.6%, 13.5%)*	45.4% (644/1417)	*+19.7% (16.2%, 23.3%)*
	Hypoxia (SpO2 < 95)	13.3% (1379/10346)	21.3% (2201/10346)	*+8.0% (6.9%, 9.0%)*	17.4% (1804/10346)	*+4.1% (3.1%, 5.1%)*	29.7% (3077/10346)	*+16.4% (15.3%, 17.5%)*	25.2% (2611/10346)	*+11.9% (10.8%, 13.0%)*
**Time to capture of events (minutes), median [IQR]**	Tachycardia (HR > 100)	37 [10, 90]	31 [8, 78]	*-6 (-15, 0)*	40 [13, 75]	*+3 (-4, 12)*	34 [15, 79]	*-3 (-10, 4)*	16 [1, 66]	*-21 (-30, -14)*
	Bradycardia (HR < 60)	54 [20, 107]	36 [13, 71]	*-17 (-24, -10)*	44 [23, 65]	*-10 (-18, -3)*	33 [19, 66]	*-21 (-14, -27)*	15 [3, 51]	*-39 (-45, -31)*
	Hypotension (MAP < 65)	15 [4, 40]	9 [3, 24]	*-6 (-9, -3)*	15 [5, 37]	*0 (-3, 6)*	14 [5, 30]	*-1 (-4, 3)*	1 [0, 5]	*-14 (-16, -11)*
	Hypoxia (SpO2 < 95)	67 [25, 132]	42 [14, 91]	*-25 (-31, -18)*	50 [24, 89]	*-17 (-23, -10)*	36 [18, 92]	*-31 (-25, -37)*	22 [3, 74]	*-45 (-50, -38)*

95% CIs derived from two-sample t-tests for mean coverage, chi-squared tests for proportions of events captured, and bootstrap resampling for time to capture.

© 2022 Kim D et al. *JAMA Network Open.*

eTable 4. Performance of Vital Sign Charting Strategies by Emergency Severity Index

		Actual		Equal spacing		Fixed schedule		80min-40min rule		Optimized	
		**ESI 2**	**ESI 3**	**ESI 2**	**ESI 3**	**ESI 2**	**ESI 3**	**ESI 2**	**ESI 3**	**ESI 2**	**ESI 3**
**VS coverage, mean (SD)**	HR	52.0% (27.6%)	51.8% (27.5%)	66.3% (24.0%)	63.6% (25.8%)	62.7% (25.2%)	61.1% (26.6%)	67.3% (24.0%)	69.1% (25.8%)	81.3% (15.8%)	80.3% (16.9%)
	MAP	51.8% (27.2%)	52.6% (28.7%)	66.4% (23.6%)	64.8% (26.6%)	61.5% (25.4%)	61.7% (27.5%)	65.7% (23.6%)	70.5% (26.6%)	80.7% (16.9%)	79.6% (18.8%)
	RR	50.9% (23.2%)	54.0% (24.1%)	60.0% (21.4%)	58.5% (23.9%)	57.4% (22.8%)	56.8% (24.6%)	60.1% (21.4%)	62.7% (23.9%)	70.9% (16.3%)	71.6% (17.0%)
	SpO2	76.2% (24.1%)	79.3% (22.6%)	80.8% (21.7%)	82.3% (21.5%)	78.5% (23.8%)	80.9% (23.1%)	82.1% (21.7%)	85.8% (21.5%)	90.1% (12.6%)	91.1% (12.0%)
**Proportion of abnormal events captured**	Tachycardia (HR > 100)	18.8% (245/1302)	14.5% (354/2448)	22.4% (291/1302)	13.1% (320/2448)	16.8% (219/1302)	10.6% (259/2448)	25.0% (325/1302)	21.9% (537/2448)	24.1% (314/1302)	16.5% (403/2448)
	Bradycardia (HR < 60)	33.1% (310/936)	27.4% (593/2168)	35.1% (329/936)	24.3% (527/2168)	27.6% (258/936)	21.5% (467/2168)	42.3% (396/936)	39.9% (864/2168)	38.9% (364/936)	29.1% (630/2168)
	Hypotension (MAP < 65)	30.0% (202/673)	16.0% (101/630)	40.3% (271/673)	25.9% (163/630)	30.8% (207/673)	18.4% (116/630)	37.0% (249/673)	35.7% (225/630)	49.6% (334/673)	37.8% (238/630)
	Hypoxia (SpO2 < 95)	16.3% (588/3615)	11.6% (750/6480)	26.5% (958/3615)	18.0% (1169/6480)	21.0% (760/3615)	15.1% (977/6480)	31.0% (1119/3615)	29.1% (1888/6480)	29.8% (1076/3615)	22.4% (1454/6480)
**Time to capture of events (minutes), median [IQR]**	Tachycardia (HR > 100)	27 [6, 78]	47 [16, 101]	28 [6, 74]	39 [13, 90]	34 [12, 78]	55 [26, 82]	34 [14, 77]	35 [16, 85]	10 [1, 60]	25 [2, 77]
	Bradycardia (HR < 60)	43 [15, 90]	63 [25, 117]	29 [10, 60]	43 [17, 80]	33 [16, 57]	54 [31, 68]	32 [18, 64]	33 [19, 67]	12 [3, 43]	18 [3, 54]
	Hypotension (MAP < 65)	17 [5, 45]	15 [6, 52]	9 [4, 23]	12 [6, 34]	16 [6, 35]	24 [7, 48]	15 [5, 32]	14 [5, 26]	1 [0, 7]	1 [0, 5]
	Hypoxia (SpO2 < 95)	54 [20, 114]	81 [31, 144]	34 [10, 84]	50 [21, 97]	36 [21, 80]	57 [35, 112]	34 [18, 89]	38 [18, 94]	17 [2, 64]	28 [4, 83]

ESI 2-3 visits account for 96.8% (24924/25751) of monitored visits. ESI = Emergency Severity Index.

© 2022 Kim D et al. *JAMA Network Open.*

eTable 5. Mean Coverage Performance of Vital Sign Charting Strategies for Emergency Severity Index Level 2 Visits With or Without Abnormalities at Triage

	Actual			Equal spacing			Fixed schedule			80min-40min rule			Optimized		
	**Triage normal**	**Triage abnormal**	***Diff (95% CI)***	**Triage normal**	**Triage abnormal**	***Diff (95% CI)***	**Triage normal**	**Triage abnormal**	***Diff (95% CI)***	**Triage normal**	**Triage abnormal**	***Diff (95% CI)***	**Triage normal**	**Triage abnormal**	***Diff (95% CI)***
**HR coverage, mean (SD)**	54.8% (27.1%)	49.0% (27.8%)	*-5.8% (-6.9%, -4.7%)*	67.7% (23.5%)	64.7% (24.5%)	*-2.9% (-3.9%, -2.0%)*	64.3% (25.1%)	61.0% (25.2%)	*-3.3% (-4.3%, -2.3%)*	68.9% (23.5%)	65.5% (24.5%)	*-3.4% (-4.2%, -2.5%)*	81.6% (15.6%)	81.1% (16.1%)	*-0.5% (-1.1%, 0.2%)*
**MAP coverage, mean (SD)**	51.7% (27.1%)	52.0% (27.3%)	*0.3% (-0.8%, 1.4%)*	65.2% (23.9%)	67.6% (23.3%)	*2.4% (1.4%, 3.3%)*	60.4% (25.6%)	62.8% (25.2%)	*2.3% (1.3%, 3.4%)*	65.1% (23.9%)	66.4% (23.3%)	*1.3% (0.4%, 2.2%)*	80.3% (17.2%)	81.1% (16.6%)	*0.8% (0.1%, 1.5%)*
**RR coverage, mean (SD)**	52.9% (23.1%)	48.6% (23.1%)	*-4.3% (-5.2%, -3.3%)*	61.0% (21.8%)	59.0% (20.9%)	*-2.0% (-2.9%, -1.1%)*	58.5% (23.1%)	56.1% (22.4%)	*-2.3% (-3.3%, -1.4%)*	61.4% (21.8%)	58.7% (20.9%)	*-2.7% (-3.5%, -1.9%)*	71.7% (16.4%)	70.0% (16.2%)	*-1.8% (-2.4%, -1.1%)*
**SpO2 coverage, mean (SD)**	79.2% (22.2%)	72.8% (25.7%)	*-6.4% (-7.4%, -5.4%)*	82.9% (20.4%)	78.4% (22.8%)	*-4.5% (-5.4%, -3.6%)*	81.3% (22.1%)	75.5% (25.2%)	*-5.8% (-6.8%, -4.9%)*	84.8% (20.4%)	79.1% (22.8%)	*-5.7% (-6.6%, -4.9%)*	91.1% (11.6%)	88.9% (13.5%)	*-2.3% (-2.8%, -1.8%)*

47.6% (4322/9089) of ESI 2 visits had one or more vital sign abnormalities at triage. 35.2% (3197/9089) were tachycardic (HR > 100), 4.7% (426/9089) were bradycardic (HR < 60), 3.2% (294/9089) were hypotensive (MAP < 65) and 11.0% (999/9089) were hypoxic (SpO2 < 95%). Differences in mean coverage reflect two-sided t-tests. Unlike other analyses of monitoring strategy performance, capture of abnormalities and time-to-capture are not shown, as patients with abnormalities at triage will by definition have a vital sign abnormality captured on initial charting.

© 2022 Kim D et al. *JAMA Network Open.*
